# Surgical Treatment Patterns, Healthcare Resource Utilization, and Economic Burden in Patients with Tenosynovial Giant Cell Tumor Who Underwent Joint Surgery in the United States

**DOI:** 10.36469/jheor.2022.32485

**Published:** 2022-03-04

**Authors:** Feng Lin, Winghan J. Kwong, Sherry Shi, Irina Pivneva, Eric Q. Wu, John A. Abraham

**Affiliations:** 1 Daiichi Sankyo, Inc.; 2 Analysis Group, Inc.; 3 Rothman Institute and Fox Chase Cancer Center

**Keywords:** tenosynovial giant cell tumors, burden of illness, treatment patterns, healthcare resource utilization, healthcare costs, absenteeism, disability

## Abstract

**Background:** Tenosynovial giant cell tumors (TGCT) are rare and locally aggressive neoplasms in synovium, bursae, and tendon sheaths, which cause pain, joint dysfunction, and damage to the affected joints.

**Objective:** To evaluate the surgical patterns and economic burden among patients with TGCT who underwent joint surgery in the United States.

**Methods:** Patients newly diagnosed with TGCT, aged 18-64 years, who underwent joint surgery post-TGCT diagnosis were identified from the OptumHealth Care Solutions, Inc database (Q1/1999-Q1/2017). Patients were required to be continuously enrolled for ≥1 year before and ≥3 years after the first TGCT diagnosis (index date). Surgical patterns were assessed post-index. Healthcare resource utilization and associated healthcare costs, and indirect costs related to work loss in year 1, year 2, and year 3 post-index, were compared with those at baseline.

**Results:** Of 835 eligible TGCT patients, 462 (55%) patients who had ≥1 joint surgery post-index were included. During a median follow-up of 5.7 years, 78% of patients underwent their first joint surgery in year 1 and 41% had ≥1 repeat surgery. Magnetic resonance imaging utilization was highest during baseline (46%) and declined afterward (28%, 17%, and 19% in years 1, 2, and 3, respectively). Opioids and nonsteroidal anti-inflammatory drugs (NSAIDs), and physical therapy, occupational therapy, and rehabilitation services, were commonly used during baseline (45%, 40%, and 30%, respectively). More patients used opioids in year 1 vs baseline (78% vs 45%; *P*<0.0001), while its utilization return to baseline levels in year 2 (41%) and year 3 (42%). A similar pattern was observed for NSAIDs and physical/occupational therapy/rehabilitation services. Healthcare resource utilization and associated healthcare costs surged in year 1 and returned to baseline or lower in years 2 and 3. A similar pattern was observed for indirect costs associated with work loss.

**Discussion:** The high proportion of patients undergoing repeat surgeries and prevalent use of opioids, NSAIDs, and physical/occupational therapy/rehabilitation services suggests an unmet medical need after surgical treatment.

**Conclusions:** Surgical resection alone might be inadequate to control TGCT. New treatment options may complement surgery and alleviate the clinical and economic burden experienced by patients with TGCT who had received prior surgery.

## INTRODUCTION

Tenosynovial giant cell tumor (TGCT) is a rare neoplastic disorder characterized by the presence of a locally aggressive proliferation of synovium of joints and tendon sheaths, which can cause pain, joint dysfunction, and damage to the joint affected.^1–3^ The condition has been shown to result from the overexpression of colony-stimulating factor 1 (CSF1) from synovial cells, which leads to an abnormal recruitment of CSF1 receptor–bearing inflammatory cells that infiltrate the synovium, causing thickening and nodularity.^4^ In the United States, TGCT is estimated to affect 1.8-9.2 per 1 million individuals.^5,6^ There are 2 types of TGCT: localized TGCT, which typically presents as a solitary nodule in or around small joints, and diffuse TGCT, which usually presents as grossly thickened synovium due to pervasive nodules infiltrating the entire synovium of larger joints.^7^ Although TGCT can affect individuals of all ages, it is most commonly documented in working-age adults and can be associated with considerable morbidity.^3,8^

Surgical resection is the primary treatment for patients with TGCT when complete tumor resection is feasible.^2,9^ Studies have shown that about half of the patients with TGCT underwent surgery.^10,11^ Although disease control may be achieved with surgery, a significant percentage of patients experience recurrence and require multiple surgical excisions, which increases the risk of complications and negative outcomes.^12,13^ A study using data from the Danish registries estimated 10-year risk of recurrence of 9.9% in localized and 19.1% in diffuse TGCT.^14^ A study reviewing patient charts from the Dutch Pathology Registry predicted reoperation rates of 17% for localized and 51% for diffuse TGCT within 5 years of initial surgery.^8^ A US claims study found 29% of patients underwent repeat operation within 2 years after the first surgery.^11^

To date, limited information is available regarding the burden of TGCT. A previous study has found that total healthcare costs for patients diagnosed with TGCT was significantly higher in the first year after compared with the year before TGCT diagnosis.^10^ In addition to having substantially higher healthcare resource use and costs, TGCT was found to associate with decreased workplace productivity as compared with patients without TGCT.^15^ While these data provide a general overview of the economic burden of TGCT, the follow-up period was short and limited to 1 year after TGCT diagnosis. In addition, data specific to the impact of TGCT surgery on healthcare resource use and workplace productivity were limited. The objective of this study was to characterize the surgical treatment patterns and the direct and indirect economic burden of surgery among patients with TGCT. Such information will be helpful to clinicians and health administrators to identify opportunities to optimize quality of care for these patients.

## METHODS

### Data Source

Data for this study were obtained from the OptumHealth Care Solutions, Inc claims database, which comprises information on over 19 million employee beneficiaries from 84 self-insured companies in the United States. Data elements include medical diagnoses and services, prescription drug claims for all beneficiaries, and disability claims for approximately 6 million beneficiaries. All data are deidentified and compliant with the Health Insurance Portability and Accountability Act. Approval from an ethics committee was not required. The dataset used in this study covers the period from January 1, 1999, to March 31, 2017.

### Study Sample and Design

Patients included in this study were required to have complete data from medical, pharmacy, and disability claims. Patients were included in the study if they (1) had at least 2 TGCT diagnoses during 2 distinct outpatient visits that were at least 30 days apart, or at least 1 TGCT diagnosis during an inpatient visit, or at least 1 TGCT diagnosis during an emergency department (ED) visit (*International Classification of Diseases, Ninth Revision, Clinical Modification* [ICD-9-CM] 727.02 and 719.2x; ICD-10-CM: D48.1, D21.0-D21.9, and M12.2x); (2) aged 18-64 years with continuous healthcare plan coverage and availability of disability data for at least 1 year before and at least 3 years after the first TGCT diagnosis; and (3) had at least 1 joint surgery, including arthroplasty, arthrodesis, arthroscopic excision, open excision, and amputation, any time after their first TGCT diagnosis.

The date of the first TGCT diagnosis was defined as the index date. The baseline period was defined as 1 year before the index date. The follow-up period was defined as the time from the index date until the end of commercial health insurance coverage, the date on which patients were eligible to receive Medicare coverage (ie, age 65 years), or end of data availability, whichever occurred first (**Supplementary Figure 1**).

### Study Measures

Surgical patterns, diagnostic procedures, concomitant medications, healthcare resource utilization, and costs were examined during the baseline and follow-up periods.

**Surgical patterns:** Joint surgeries, including arthroplasty, arthrodesis, arthroscopic excision, open excision, and amputation were described using frequencies and proportions. The timing of the first surgery and repeat surgery post-index was assessed by year following the first TGCT diagnosis (≤1 year [year 1]; >1 year and ≤2 years [year 2]; >2 years and ≤3 years [year 3]; and >3 years [year 4+]).

**Healthcare resource utilization and associated direct healthcare costs:** All-cause healthcare resource utilization categorized by the place of service (inpatient, outpatient, ED, and pharmacy) and their associated costs were evaluated from the payer’s perspective at baseline and in the years 1, 2, and 3 post-index, separately. Diagnostic tests, concomitant medication use, supportive therapies, and visits to specialists, available in the data as provider specialty, were described. All costs were adjusted to 2018 US dollars (USD) using the medical care component of the Consumer Price Index.

**Indirect economic burden:** The indirect economic burden of TGCT included time lost from work due to disability and time lost from work due to medical visits, and their associated costs from the employer’s perspective. Time lost from work due to disability was defined as the duration of absence from work while receiving short- or long-term disability benefits. Time lost from work related to medical visits was estimated based on the following assumptions: (1) patients missed 1 day of work for each weekday with an ED claim or for each weekday during an inpatient stay, and (2) patients missed a half day of work on each weekday with an outpatient visit. To avoid double counting, disability claims were prioritized over medical visits, and inpatient/ED visits were prioritized over outpatient visits, if they occurred on the same day. Costs associated with absenteeism due to medical visits were computed by multiplying the number of days lost from work due to medical visits by an employee’s daily wage (converted from the employee’s yearly wage at the index date, as reported in the data) and adjusted to 2018 USD using the Hourly Compensation Index. Short- and long-term disability costs were based on an employee’s disability claims.

**Statistical analysis:** Continuous variables were summarized using means, medians, and SD; categorical variables were summarized using frequencies and proportions. Joint surgeries, concomitant medications, procedures, and healthcare resource utilization in year 1, year 2, and year 3 post-index were compared with those at baseline using Wilcoxon signed-rank tests for continuous variables and McNemar tests for categorical variables.

## RESULTS

### Baseline Characteristics

**Figure 1.**
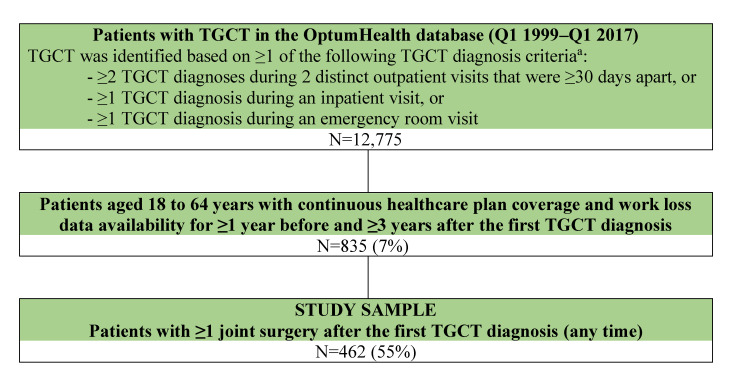
Selection of Study Sample Abbreviations: Q, quarter; TGCT, tenosynovial giant cell tumor. ^a^ TGCT diagnosis codes: *International Classification of Diseases, Ninth Revision, Clinical Modification* (ICD-9-CM): 719.2x, 727.02; ICD-10-CM: D48.1, D21.0-D21.9, M12.2.

Of 835 patients with TGCT and at least 3 years of follow-up, 462 (55%) patients who had at least one joint surgery post-index were included in the study sample (Figure 1). The median age of the studied cohort was 47.0 years, and 34% were female (Table 1). Patients were from all census regions in the United States, with a moderate preponderance of patients from the South (44%). Nearly half the patients were part of a preferred provider organization. At baseline, the mean (SD) Charlson Comorbidity Index score was 0.3 (0.7); the most prevalent comorbidities were hypertension (25%) and depression (9%). A minority of patients (8%) underwent joint surgery in the year prior to their first TGCT diagnosis. Baseline characteristics of the studied cohort (ie, the surgical cohort) were similar to that of patients with at least 3 years of follow up but who had not undergone surgery (ie, the nonsurgical cohort; **Supplementary Table 1**).

**Table 1.** Baseline Characteristics

** **	**Study Sample (n=463)**
Age at index date (years), mean [median]	45.9 [47.0]
18-24 years, n (%)	8 (1.7)
25-40 years, n (%)	102 (22.1)
41-64 years, n (%)	352 (76.2)
Female, n (%)	155 (33.5)
US region, n (%)	
South	201 (43.5)
Northeast	141 (30.5)
Midwest	68 (14.7)
West	50 (10.8)
Unknown	2 (0.4)
Type of healthcare plan, n (%)	
Preferred provider organization	220 (47.6)
Point-of-service	140 (30.3)
Other^a^	102 (22.1)
Year of index date, n (%)	
2000-2005	189 (40.9)
2006-2010	208 (45.0)
2011-2016	65 (14.1)
Annual employee earnings at the index date (2018 US$), mean [median]	69 907 [55 668]
Employer industry, n (%)	
Transportation	198 (42.9)
Manufacturing	107 (23.2)
Technology	47 (10.2)
Telecommunications	25 (5.4)
Energy/utility	22 (4.8)
Healthcare	15 (3.2)
Government/religion/education services	5 (1.1)
Financial services/insurance	4 (0.9)
Retail/consumer goods	4 (0.9)
Food/beverage	2 (0.4)
Other service	33 (7.1)
Charlson Comorbidity Index,^b^ mean±SD	0.3±0.7
0, n (%)	383 (82.9)
1, n (%)	44 (9.5)
≥2, n (%)	35 (7.6)
Comorbidities with prevalence ≥1%,^b^ n (%)	
Hypertension	114 (24.7)
Depression	40 (8.7)
Hypothyroidism	32 (6.9)
Chronic pulmonary disease	32 (6.9)
Diabetes without chronic complication	23 (5.0)
Any malignancy (including blood, excluding skin)	17 (3.7)
Valvular disease	17 (3.7)
Cardiac arrhythmias	14 (3.0)
Fluid and electrolyte disorders	12 (2.6)
Obesity	11 (2.4)
Rheumatic disease	11 (2.4)
Cerebrovascular disease	7 (1.5)
Deficiency anemia	7 (1.5)
Mild liver disease	7 (1.5)
Renal disease	7 (1.5)
Congestive heart failure	5 (1.1)
Diabetes with chronic complication	5 (1.1)
Drug abuse	5 (1.1)
Peripheral vascular disease	5 (1.1)
Patients with joint surgery in the baseline year,^c^ n (%)	38 (8)

^a^ Health maintenance organization, any indemnity, locked-in, and exclusive provider organization.

^b^ Measured based on diagnosis codes in the claims data in the baseline year.

^c^ Joint surgeries, including arthroplasty, arthrodesis, arthroscopic excision, open excision, and amputation, occurred within 12 months prior to the first TGCT diagnosis.

### Surgical Patterns

**Figure 2.**
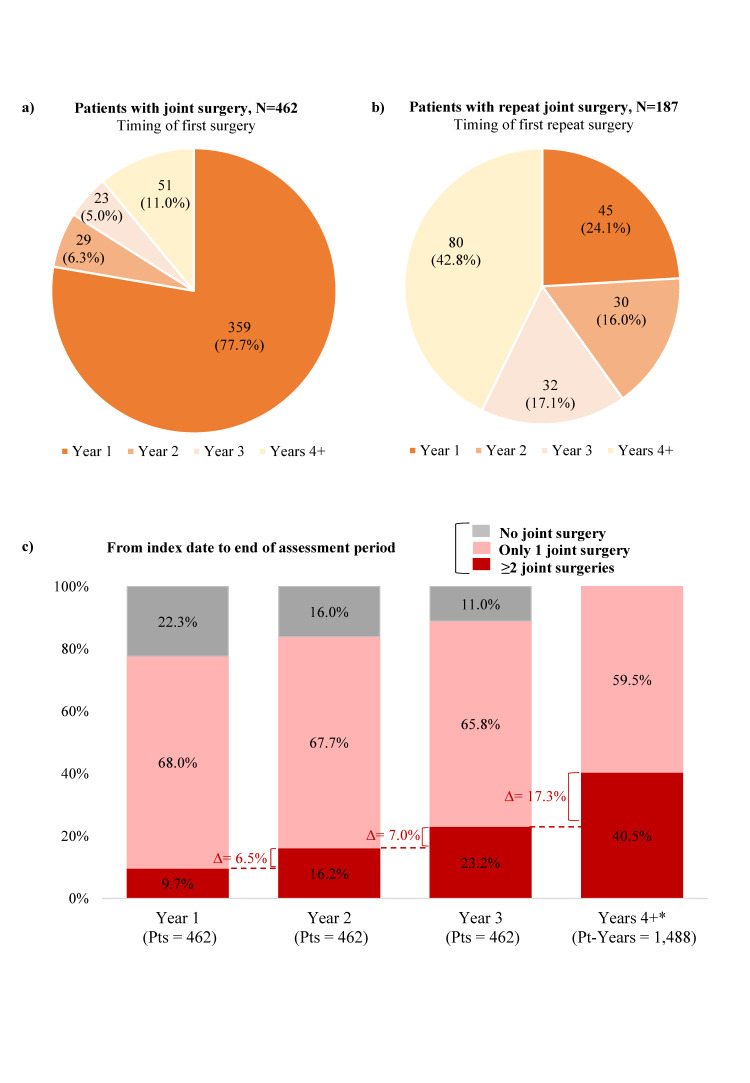
Surgical Patterns: (A) Distribution of First Surgeries Post-TGCT Diagnosis; (B) Distribution of Repeat Joint Surgeries Post-TGCT Diagnosis; (C) Cumulative Distribution of the Number of Joint Surgeries^a^ During Follow-up Abbreviations: Pts, patients; Years 1, 2, 3, first, second, and third year post-index, respectively (by design, patients had ≥3 years of follow-up); Year 4+, all subsequent years post-index (may differ between patients). ^a^ Surgery more than 30 days after a previous joint surgery was defined as a separate surgery. * Given follow-up varies across patients starting with year 4+, we present patient-years beyond year 4 instead of patients.

During the median follow-up of 5.7 years (mean, 6.3; range, 3-15.2), 78% of patients received first joint surgery in year 1, 6% in year 2, 5% in year 3, and 11% in subsequent years (Figure 2A). The median time from the index diagnosis to the first joint surgery was 25 days, although 103 patients did not have surgery until year 2 or later.

A total of 187 (41%) patients had at least one repeat surgery; the mean (SD) number of repeat surgeries for these patients was 1.73 (1.18). Among patients receiving repeat surgery, most patients had their first repeat surgery in year 1 (43%; Figure 2B). The cumulative proportion of patients who had repeat surgery by the end of years 1, 2, and 3 was 10%, 16%, and 23%, respectively (Figure 2C). Among the 359 patients who had their first surgery in year 1, 43% had repeat surgeries (13% in year 1, 8% in year 2, 7% in year 3, and 15% after year 3).

### Healthcare Resource Utilization and Associated Direct Healthcare Costs

Magnetic resonance imaging (MRI) was performed in 46% of patients at baseline, and its utilization significantly declined during the 3 years following the index TGCT diagnosis (year 1, 28%; year 2, 17%, year 3, 19%, all *P*<0.0001; Table 2]). Opioids and nonsteroidal anti-inflammatory drugs (NSAIDs) were the most commonly used medications at baseline (45% and 40%, respectively). Opioid and NSAID utilization increased significantly to 78% and 49%, respectively, in year 1 (all *P*<0.001). In year 2 and year 3, their utilization declined below baseline levels. The use of systemic corticosteroids remained stable at 18%-19% throughout the study period. Although a small proportion of patients used disease-modifying antirheumatic drugs (baseline, 3%; year 1, 4%; year 2, 5%; year 3, 5%), a statistically significant increase in their use was observed from baseline to year 3 (from 3% to 5%; *P*=0.0253).

**Table 2.** Healthcare Resource Utilization in Patients with TGCT Who Underwent Joint Surgery

**Healthcare Resource Utilization**	**Study Sample (n = 462)**			
	**Baseline Year**	**Year 1**	**Year 2**	**Year 3**
Patients with MRI, n (%)	210 (46)	128 (28)*	78 (17)*	87 (19)*
Patients with prescriptions for selected medications, n (%)				
Opioid	209 (45)	359 (78)*	187 (41)	196 (42)
NSAIDs	184 (40)	226 (49)*	164 (36)	149 (32)*
Systemic corticosteroids	83 (18)	86 (19)	82 (18)	88 (19)
DMARDs	15 (3.2)	20 (4.3)	22 (4.8)	25 (5.4)*
Patients with supportive care services, n (%)				
Physical and/or occupational therapy/rehabilitation	138 (30)	268 (58)*	132 (29)	137 (30)
Chiropractic	66 (14)	58 (13)	61 (13)	71 (15)
Acupuncture	3 (1)	7 (2)	8 (2)	9 (2)
Osteopathic	9 (2)	8 (2)	8 (2)	7 (2)
Patients with healthcare visits by visit setting, n (%)				
Inpatient admissions^a^	55 (12)	113 (25)*	43 (9)	44 (10)
ED visits	111 (24)	130 (28)	94 (20)	103 (22)
Outpatient visits	452 (98)	461 (100)*	434 (94)*	443 (96)
Number of visits/patient by visit setting, mean±SD [median]				
Inpatient admissions^a^	0.4±1.9 [0]	0.7 ±1.9 [0]*	0.4±2.0 [0]	0.4±3.2 [0]
ED visits	0.4±0.9 [0]	0.6±2.0 [0] *	0.4±1.5 [0]	0.4±1.5 [0]
Outpatient visits	16.5±18.4 [11]	24.2±20.3 [18]*	15.3±17.8 [10]*	14.9±15.4 [10]*
Patients with healthcare visits by specialist type, n (%)				
Surgeon	396 (86)	410 (89)*	238 (52)*	217 (47)*
Nonsurgical orthopedist^b^	62 (13)	65 (14)	50 (11)	47 (10)
Rheumatologist	20 (4)	31 (7)*	22 (5)	17 (4)
Oncologist	4 (1)	5 (1)	4 (1)	10 (2)

Abbreviations: DMARD, disease-modifying antirheumatic drug; ED, emergency department; MRI, magnetic resonance imaging; NSAIDs, nonsteroidal anti-inflammatory drugs; TGCT, tenosynovial giant cell tumor; Years 1, 2, 3, first, second, and third year post-index, respectively (by design, patients had ≥3 years of follow-up).

* Statistically significant compared with baseline value (ie, *P*<0.05 using McNemar tests for proportions and Wilcoxon signed rank-sum tests for for counts).

^a^ Inpatient admissions did not include same-day surgeries, which were classified as outpatient visits.

^b^ Included podiatrists and other orthopedic specialists (eg, sports medicine).

Surgeons were the most visited specialists at baseline and year 1 (89% vs 86%; *P*=0.0308). About half of patients had office visits to surgeons in subsequent years (52% in year 2 and 47% in year 3 vs 86% in the baseline year; all *P*<0.0001).

About one-third of the patients used physical therapy, occupational therapy, or rehabilitation services at baseline. More patients used supportive services in year 1 (58%, *P*<0.0001), while no significant differences were observed in year 2 and year 3 relative to baseline. The use of chiropractic services remained stable at 13%-15% in the baseline year and the 3 years post-index. Osteopathic services were rarely used in all periods.

Compared with baseline, patients had significantly higher rates of inpatient admissions (0.7 vs 0.4 per patient year [PPY]; *P*<0.0001), ED visits (0.6 vs 0.4 PPY; *P*=0.0043), and outpatient visits (24.2 vs 16.5 PPY; *P*<0.0001) in year 1. Compared with baseline, the average rate of outpatient visits was significantly reduced in year 2 (15.3 vs 16.5 PPY; *P*=0.0108) and year 3 (14.9 vs 16.5 PPY; *P*=0.0126). No significant differences in inpatient admissions or ED visits were observed beyond year 1.

**Figure 3.**
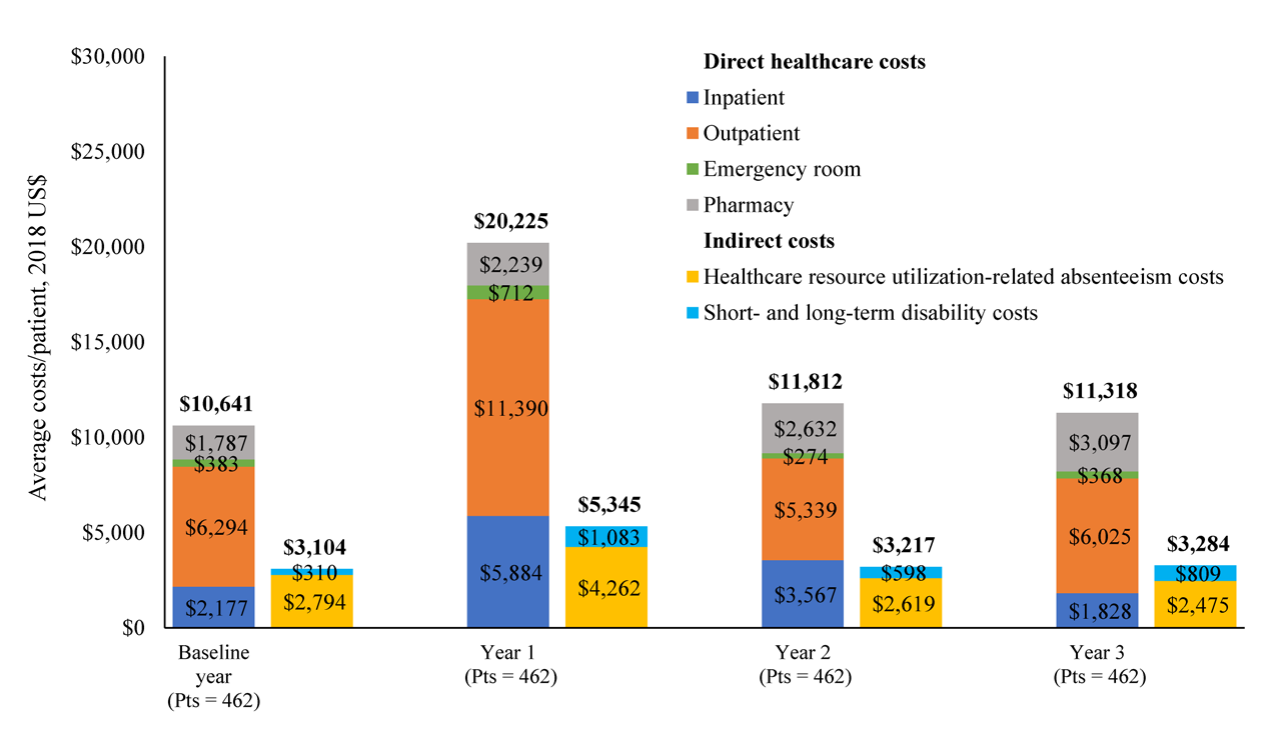
All-Cause Direct Healthcare Costs and Indirect Costs Abbreviation: Pts, patients.

The mean all-cause healthcare costs were higher in year 1 compared with baseline (mean: $20 225 vs $10 641 PPY) and returned to baseline level in year 2 ($11 812 PPY) and year 3 ($11 318 PPY). More than half the cost was driven by outpatient visits (Figure 3).

### Indirect Economic Burden

In the baseline year, 3% of patients had records of disability claims, and the mean time loss from work was 7.9 days (**Supplementary Table 2**). The proportion of patients with disability claims was highest during year 1 (9%), with a return to baseline level in subsequent years (5% for years 2 and 3). The corresponding mean total indirect costs were highest during year 1 ($5345 PPY), with costs decreased to baseline levels in years 2 and 3 ($3217 and $3284 PPY, respectively).

## DISCUSSION

Although surgery is the first-line treatment for TGCT, only half of TGCT patients identified in this analysis received surgical resection after their first TGCT diagnosis. This finding is consistent with findings reported by other retrospective analyses.^10,11,14^ Surgical procedures usually occurred soon after index diagnosis (median time to first surgery, 25 days), but 11% of the patients did not receive surgery until 3 years later. It is possible that patients with manageable symptoms might choose the “wait and see” approach, while patients with severely impaired morbidity might consider earlier intervention to preserve joint function and improve functionality.

Over 40% of patients underwent one or more repeat surgeries, suggesting a single surgical resection alone may be inadequate to control TGCT for many patients. Although surgery may be curative for some patients, complete removal of diffuse tumor is challenging, and the tumor can often grow back after removal. Lifetime recurrence rates for localized TGCT could be up to 15%, and recurrence rates for diffuse TGCT were estimated to be 20%-50%.^2,13,14,16,17^ Our finding was consistent with results of another claims analysis, which reported the risk of repeated surgery to be 21.5% at 1 year and 29.2% at 2 years after the first surgery among TGCT patients.^11^ In addition to removal of recurrent tumor growth, joint arthroplasty or amputation might be required to restore mechanical joint function.^18,19^

Supportive care to surgery, such as utilization of pain management and rehabilitation services, was highest in the first year post-diagnosis, when most surgeries were observed. Although MRI is the most distinctive diagnostic tool, decreased utilization of MRI in the years following surgery suggests MRI was not commonly used to detect recurrence. Utilization of opioids and NSAIDs, physical/occupational/rehabilitation services, and outpatient visits with surgeons remained prevalent more than 2 years after TGCT diagnosis. Similar to findings of other claims studies on TGCT patients, outpatient visits represented the main driver of healthcare costs in the year following TGCT diagnosis.^10,15^ The increase in healthcare costs in year 1 from baseline was 90%, which was higher than the magnitude of increase reported in previous studies where both surgical and nonsurgical TGCT patients were included (35%).^10^ Corresponding to the time trend in healthcare resource utilization, work productivity loss related to absenteeism and disability was also highest in year 1, when most surgeries occurred. Although the findings on absenteeism and disability provide some insights into the impact of surgery on patients, future studies are warranted to better understand treatment outcomes and quality of care following first and/or repeated surgery from the patients’ perspectives.

Collectively, findings of this study underscore the increased direct and indirect burden associated with surgery for TGCT, high level of postsurgical supportive care required, and continued use of concomitant medications, such as opioids. Importantly, over 40% of surgical patients required at least one repeat surgery, implying the high risk of recurrence even after receiving surgery, the mainstay treatment of TGCT. Not only do repeated surgeries increase the risk of complications and infection, the continued inflammation and bone involvement may lead to articular destruction, which can cause secondary osteoarthritis. All these factors contributed to the increased burden of TGCT following surgery. To this end, novel, nonsurgical treatments may provide options to alleviate some of these burdens. Pexidartinib was the first systemic therapy approved in the United States for TGCT, based on the pivotal ENLIVEN trial, which demonstrated a 53% overall response rate and meaningful improvements in physical function and stiffness in patients with advanced TGCT not amenable to improvement with surgery.^20–22^ Additional pharmacological therapies under investigation include other CSF1 receptor inhibitors, anti-CSF1 receptor monoclonal antibodies, tyrosine kinase inhibitors, and NSAIDs. These novel therapies have the potential for use as a primary treatment or as part of a multimodal therapy (eg, as adjuvant therapy) for TGCT to prevent recurrence and mitigate the clinical and economic burden associated with surgery.^1,22^

The current study is subject to several limitations. First, because medical claims are used for payment purposes, diagnosis codes are subject to errors and miscoding. Without detailed clinical information, it is difficult to determine histological type (localized vs diffuse), tumor location, or severity of TGCT to assess the clinical rationale and outcomes for surgical treatment. Second, although patient perspective is a critical part in TGCT management, patient-reported outcomes, which are collected using questionnaires, are typically not integrated with claims data. As TGCT and surgery would affect patient functional status, future studies on patient-reported outcomes will complement the current data to further the understanding of the indirect economic burden of surgical TGCT treatment. Third, the study sample was limited to privately insured employees aged 65 years or younger who remained employed and underwent surgery. Findings may not be generalized to other TGCT patients, especially those uninsured or covered by Medicaid, or those who did not undergo surgery during the study period. Lastly, the assumption on time lost from work due to medical visits is an approximation used in prior work^23^ and needs further validation.

## CONCLUSIONS

Despite being the standard treatment option, joint surgeries alone might be inadequate to control TGCT for patients who experience tumor recurrence. The increases in healthcare resource utilization, healthcare costs, and work productivity loss were the most pronounced in the first year post-diagnosis, when most surgeries were observed. Utilization of opioids and NSAIDs, physical/occupational/rehabilitation services, and outpatient visits with surgeons remained prevalent more than 2 years after TGCT diagnosis. The findings highlight challenges for both physicians and the healthcare system in the diagnosis and treatment of TGCT. Although surgery remains the most common treatment for TGCT, there may be an increasing role for novel therapies, including targeted agents, which may help reduce the clinical and economic burden associated with the surgical treatment. Weighing the side effects of these therapies against the symptomatic benefit on a patient-by-patient basis in TGCT remains critical.

### Disclosures

FL and WJK are employees of Daiichi Sankyo Inc and may own stocks/stock options; FL additionally declares owning stocks of Novartis. SS, IP, and EQW are employees of Analysis Group Inc, which provided paid consulting services to Daiichi Sankyo Inc for the conduct of this study. JAA declares receiving research funding from Janssen Oncology and Novartis.

### Meeting Presentation

Part of the material in this manuscript was presented at the Connective Tissue Oncology Society (CTOS) 2020 Annual Meeting (virtual).

## Supplementary Material

Supplementary Online Material
