# Author Correction: Surgical Treatment Patterns, Healthcare Resource Utilization, and Economic Burden in Patients with Tenosynovial Giant Cell Tumor Who Underwent Joint Surgery in the United States

**DOI:** 10.26469/jheor.2022.33289

**Published:** 2022-03-16

**Authors:** Feng Lin, Winghan J. Kwong, Sherry Shi, Irina Pivneva, Eric Q. Wu, John A. Abraham

**Affiliations:** 1 Daiichi Sankyo, Inc.; 2 Analysis Group, Inc.; 3 Rothman Institute and Fox Chase Cancer Center

**Keywords:** tenosynovial giant cell tumors, burden of illness, treatment patterns, healthcare resource utilization, healthcare costs, absenteeism, disability

Correction to: https://doi.org/10.36469/jheor.2022.32485

Published online: March 3, 2022

We have identified a discrepancy in the results section under surgical patterns between a value reported in the text and what is depicted in Figure 2B. The original publication stated that most of the patients (43%) receiving repeat surgery had their first repeat surgery in year 1. However, this text did not include a supporting figure to represent findings for this specific statement (eg, rolling window of concomitant surgeries in the same year or the neighboring years). Based on what is represented in Figure 2B across several years, the text in the results should read:

Among patients receiving repeat surgery, about a quarter of patients had their first repeat surgery in year 1 (24%; Figure 2B).

This error has no effect on the interpretation and conclusion of the results. The downloadable article has been revised to reflect this correction.

