# Supplementary material for: Surgical Treatment Patterns, Healthcare Resource Utilization, and Economic Burden in Patients with Tenosynovial Giant Cell Tumor Who Underwent Joint Surgery in the United States
Source: J Health Econ Outcomes Res. 2022 Mar 4;9(1):68–74. doi: 10.36469/jheor.2022.32485 (PMC8896882; doi:10.36469/jheor.2022.32485)
Supplement: Supplementary Online Material [file jheor_2022_9_1_32485_83772.pdf]

Journal of Health Economics and Outcomes Research

Online Supplementary Material

Surgical Treatment Patterns, Healthcare Resource Utilization, and Economic Burden in Patients with Tenosynovial Giant Cell Tumor Who Underwent Joint Surgery in the United States. *JHEOR*. **2022;9(1):68-74.** doi:10.36469/jheor.2022.32485

Figure S1: Study Design

Table S1: Baseline Characteristics of Unmatched Nonsurgical Cohort vs Surgical Cohort

Table S2: All-Cause Work Loss and Associated Indirect Costs

This supplementary material has been provided by the authors to give readers additional information about their work.

This is an open-access article distributed under the terms of the Creative Commons Attribution 4.0 International License (CCBY-4.0). View this license's legal deed at http://creativecommons.org/licenses/by/4.0 and legal code at http://creativecommons.org/licenses/by/4.0/legalcode for more information.

Table S1. Study Design

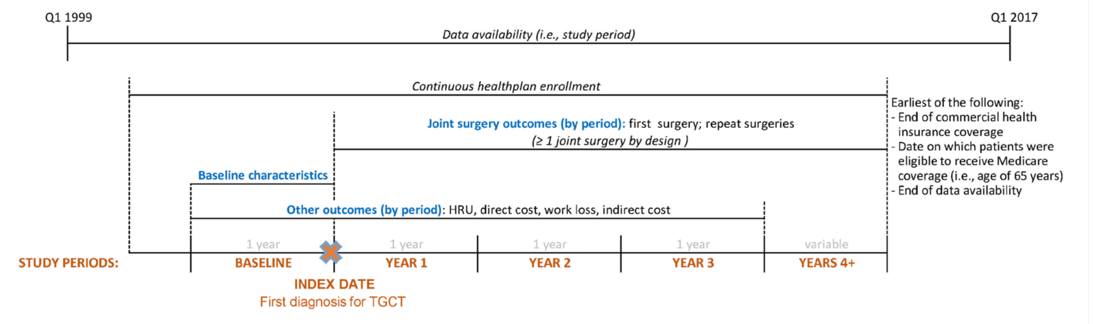

Abbreviations: ICD-9-CM, *International Classification of Diseases, Ninth Revision, Clinical Modification;* ICD-10-CM, *International Classification of Diseases, Tenth Revision, Clinical Modification;* Q, quarter; TGCT, tenosynovial giant cell tumors.

TGCT diagnosis codes: ICD-9-CM: 719.2x, 727.02; ICD-10-CM: D48.1, D21.0-D21.9, M12.2.

Table S1. Baseline Characteristics of Unmatched Nonsurgical Cohort vs Surgical Cohort

	**Nonsurgical Cohort (n=373)**	**Surgical Cohort (n=462)**
Age at index date, mean [median]	45.8±8.9 [47.0]	45.9 [47.0]
18-24 years, n (%)	7 (1.9)	8 (1.7)
25-40 years, n (%)	86 (23.1)	102 (22.1)
41-64 years, n (%)	280 (75.1)	352 (76.2)
Female, n (%)	129 (34.6)	155 (33.5)
US region, n (%)		
South	175 (46.9)	201 (43.5)
Northeast	97 (26.0)	141 (30.5)
Midwest	57 (15.3)	68 (14.7)
West	44 (11.8)	50 (10.8)
Unknown	0 (0.0)	2 (0.4)
Type of healthcare plan, n (%)		
Preferred provider organization	182 (48.8)	220 (47.6)
Point-of-service	123 (33.0)	140 (30.3)
Other[a]	68 (18.3)	102 (22.1)
Year of index date, n (%)		
2000-2005	131 (35.1)	189 (40.9)
2006-2010	163 (43.6)	208 (45.0)
2011-2016	79 (21.1)	65 (14.1)
Annual employee earnings at the index date (2018 US$), mean [median]	69 026 [53 601]	69 907 [55 668]
Employer industry, n (%)		
Transportation	179 (48.0)	198 (42.9)
Manufacturing	72 (19.3)	107 (23.2)
Technology	27 (7.2)	47 (10.2)
Telecommunications	24 (6.4)	25 (5.4)
Energy/utility	18 (4.8)	22 (4.8)
Healthcare	14 (3.8)	15 (3.2)
Government/religion/education services	4 (1.1)	5 (1.1)
Financial services/insurance	5 (1.3)	4 (0.9)
Retail/consumer goods	5 (1.3)	4 (0.9)
Food/beverage	3 (0.8)	2 (0.4)
Other service	22 (5.9)	33 (7.1)
Charlson Comorbidity Index,[b] mean±SD	0.3±0.8 [0.0]	0.3±0.7
0, n (%)	319 (85.5)	383 (82.9)
1, n (%)	33 (8.8)	44 (9.5)
≥ 2, n (%)	21 (5.6)	35 (7.6)
Comorbidities with prevalence ≥1%,[b] n (%)		
Hypertension	83 (22.3)	114 (24.7)
Depression	28 (7.5)	40 (8.7)
Hypothyroidism	26 (7.0)	32 (6.9)
Chronic pulmonary disease	19 (5.1)	32 (6.9)
Diabetes without chronic complication	23 (6.2)	23 (5.0)
Any malignancy (including blood, excluding skin)	11 (2.9)	17 (3.7)

Valvular disease	15 (4.0)	17 (3.7)
Cardiac arrhythmias	10 (2.7)	14 (3.0)
Fluid and electrolyte disorders	7 (1.9)	12 (2.6)
Obesity	9 (2.4)	11 (2.4)
Rheumatic disease	19 (5.1)	11 (2.4)
Cerebrovascular disease	2 (0.5)	7 (1.5)
Deficiency anemia	6 (1.6)	7 (1.5)
Mild liver disease	8 (2.1)	7 (1.5)
Renal disease	1 (0.3)	7 (1.5)
Congestive heart failure	3 (0.8)	5 (1.1)
Diabetes with chronic complication	1 (0.3)	5 (1.1)
Drug abuse	1 (0.3)	5 (1.1)
Peripheral vascular disease	7 (1.9)	5 (1.1)
Patients with joint surgery in the baseline year,[c] n (%)	—	38 (8)

[a]Health maintenance organization, any indemnity, locked-in, and exclusive provider organization.
[b]Measured based on diagnosis codes in the claims data in the baseline year.
[c]Joint surgeries, including arthroplasty, arthrodesis, arthroscopic excision, open excision, and amputation, occurred within 12 months prior to the first TGCT diagnosis.

Table S2. All-Cause Work Loss and Associated Indirect Costs

All-Cause Indirect Burden	Study Sample (n=462)			
	**Baseline Year**	**Year 1**	**Year 2**	**Year 3**
Patients with any absenteeism, n (%)	422 (91.3)	428 (92.6)	403 (87.2)	412 (89.2)
Patients with any disability, n (%)	14 (3.0)	43 (9.3)	21 (4.5)	24 (5.2)
Total work loss days, mean [median]	9.9 [6]	19.4 [11]	12.7 [6]	12.9 [5]
Healthcare resource utilization–related absenteeism days	7.9 [6]	12.2 [10]	7.8 [5]	7.6 [5]
Short- and long-term disability days	1.9 [0]	7.2 [0]	4.9 [0]	5.3 [0]
Total indirect costs (2018 US$), mean [median]	3104 [1533]	5345 [2902]	3217 [1520]	3284 [1531]
Healthcare resource utilization–related absenteeism costs	2794 [1485]	4262 [2639]	2619 [1387]	2475 [1433]
Short- and long-term disability costs	310 [0]	1083 [0]	598 [0]	809 [0]

Abbreviation: US$, United States dollars.
